# Late Established Mutans Streptococci in Children over 3 Years Old

**DOI:** 10.1155/2010/732468

**Published:** 2010-02-14

**Authors:** Mitsugi Okada, Yoshiko Taniguchi, Fumiko Hayashi, Takako Doi, Junji Suzuki, Motoyuki Sugai, Katsuyuki Kozai

**Affiliations:** ^1^Department of Special Care Dentistry, Hiroshima University Hospital, 1-2-3, Kasumi, Minami-ku, Hiroshima 734-8553, Japan; ^2^Department of Pediatric Dentistry, Hiroshima University Hospital, 1-2-3, Kasumi, Minami-ku, Hiroshima 734-8553, Japan; ^3^Department of Pediatric Dentistry, Graduate School of Biomedical Sciences, Hiroshima University, 1-2-3, Kasumi, Minami-ku, Hiroshima 734-8553, Japan; ^4^Department of Bacteriology, Graduate School of Biomedical Sciences, Hiroshima University, 1-2-3, Kasumi, Minami-ku, Hiroshima 734-8553, Japan

## Abstract

Acquisition of mutans streptococci has been reported to most commonly occur at approximately 26 months of age. In the present study, we detected *Streptococcus mutans* and *S. sobrinus* using polymerase chain reaction (PCR) assays in children, then re-examined the subjects to determine the time of acquisition of these bacteria over a 1-year period. The subjects were 57 children ranging in age from 3 to 5 years old, each with primary dentition. Plaque samples were collected from all erupted tooth sites using a sterile toothbrush. PCR assays were performed to detect the targeted mutans streptococci at the beginning of the study (baseline) and after 1 year. At the baseline examination, the prevalence of *S. mutans* and *S. sobrinus* was 61.4% and 54.4%, respectively, in all subjects, of whom 14 (24.6%) were positive for *S. mutans* alone, 10 (17.5%) for *S. sobrinus* alone, and 21 (36.8%) for both *S. mutans* and *S. sobrinus*, with 12 (21.1%) negative for both. After 1 year, 4 of 22 (18.2%) subjects newly had acquired *S. mutans* and 15 of 26 (57.7%) had aquired *S. sobrinus*, while 5 (8.8%) remained negative for both bacteria. The age of the first positive *S. mutans* finding ranged from 49 to 71 months, while that for *S. sobrinus* ranged from 49 to 81 months old. Our results suggest that *S. sobrinus* becomes established later than *S. mutans* in the oral cavities of children over the age of 3 years old.

## 1. Introduction

Mutans streptococci, comprised of *Streptococcus mutans* and *S. sobrinus*, are considered to be the principal etiologic agents of dental caries in humans [1–3]. Preschool children harboring both *S. mutans* and *S. sobrinus* have a significantly higher incidence of dental caries than those with *S. mutans* alone [4]. Some studies have suggested that there is a “window of infectivity” for mutans streptococci at an early age, after which colonization is not likely to occur [5–7], and others have reported its predentate presence in infants as young as 3 months of age [8–10]. It was reported that 10 of 15 children acquired mutans streptococci during a 7-year period, and a second “window of infectivity” after the age of 5 when the permanent dentition erupts has been postulated [11]. Although several studies have attempted to determine the time of initial mutans streptococci acquisition, it remains controversial.

In several epidemiologic studies, identification of *S. mutans* and *S. sobrinus* on such selective media as mitis-salivarius (MS) or MS-bacitracin (MSB) agar has been performed using colonial morphology methods [12–14]. However, accurate differentiation between *S. mutans* and *S. sobrinus* is not easy, as well as time-consuming and laborious [6], and it has been reported that *S. sobrinus* from dental plaque samples is especially difficult to culture directly on MSB selective medium [15, 16]. Thus, it is of great importance to distinguish the presence of these 2 species separately in children for accurate prediction and effective prevention of dental caries.

Thus far, several methods used for detecting and identifying mutans streptococci, including direct microscopy, cultivation, enzyme tests, monoclonal antibodies, enzyme-linked immunosorbent assays, and species-specific DNA probes, have been reported [17–20]. Several investigators have also developed polymerase chain reaction (PCR) methods and found them to be more sensitive for detection, when compared to conventional culture techniques [21–23], as they have been shown able to detect low numbers of bacterial species with a detection limit of as few as 25–100 cells [22–24], while being quick and relatively simple to perform. Further, PCR assays have been reported suitable for the specific detection and identification of human cariogenic bacteria, such as *S. mutans* and *S. sobrinus* [22–25].

 In the present study, we detected *S. mutans* and *S. sobrinus* using polymerase chain reaction (PCR) assays in preschool children, then re-examined the subjects to determine acquisition of those bacteria over a 1-year period.

## 2. Materials and Methods

Fifty-seven Japanese preschool children, aged 3 to 5 years old with primary dentition, who were visitors to Hiroshima University Hospital, were enrolled. Consent for participation was obtained from at least one of their parents prior to the study according to the ethical guidelines of the Declaration of Helsinki (1975). Those who had received antibiotics within the previous 3 months or with systemic diseases were excluded. 

### 2.1. Plaque Sampling

Dental plaque samples at the beginning (baseline) of the study and after 1 year were collected from all erupted teeth by professionally brushing with a sterile toothbrush for 1 minute, using a previously described method [26]. Plaque adhering to the toothbrush was removed by washing several times in a tube of sterile distilled water. The plaque samples were immediately transported to our research laboratory and stored at −20°C, prior to extraction of genomic DNA. One year later, plaque samples were again obtained from the same subjects in the same manner.

### 2.2. Genomic DNA Preparation

*Streptococcus mutans* JCM5175 and *S. sobrinus* ATCC27607 were used as control organisms. PCR detection of the tested species was performed using primers, as previously described [22, 24], while that of 16S ribosomal RNA encoding gene (GenBank accession number M75035) was used as previously described [27].

Plaque samples were first harvested by centrifugation at 1,600× g for 20 minutes. The supernatants were discarded, and the individual cell pellets were stored at −20°C until DNA isolation. A genomic DNA preparation from each plaque sample was obtained using a standard miniprep procedure [28], to which we added an RNase treatment [29]. DNA concentrations in the dental plaque samples were calculated by measuring A260 and the quality was estimated by the A260/A280 ratio [30].

### 2.3. Conditions of PCR Amplification

PCR amplification was performed in a reaction mixture (25 *μ*L) consisting of PCR beads (GE Healthcare UK Limited, UK) that contained an enzyme (Taq DNA polymerase), along with the required reagents, 25 pmol of each primer, and 20 to 50 ng of template DNA solution in a thermal cycler (PC-700 program temp control system, ASTEC Co. Ltd., Fukuoka, Japan). Each set of PCR analyses included a negative control (water blank) in addition to the positive control. The reaction mixture was denatured at 95°C for 3 minutes, followed by a series of amplification-denaturation steps at 95°C for 1 minute, annealing at 55°C for 1 minute, and extension at 72°C for 1 minute, which was repeated for 26 cycles, with a final cycle at 94°C for 1 minute, 55°C for 1 minute, and 72°C for 5 minutes [22]. Following amplification, 15 *μ*L of the PCR products were analyzed by electrophoresis on a 1.2% agarose gel. After staining with ethidium bromide, the newly synthesized DNA fragments were visualized under a 302-nm ultraviolet light. The size of the PCR products was estimated from the electrophoretic migration of products relative to a 100 base-ladder marker (Amersham Pharmacia Biotech, AB, Uppsala, Sweden).

## 3. Results

Table 1 shows the distribution of mutans streptococci at the baseline examination. The prevalence of *S. mutans* and *S. sobrinus* was 61.4% and 54.3%, respectively, in all subjects, of whom 14 (24.6%) were positive for *S. mutans* alone, 10 (17.5%) for *S. sobrinus* alone, and 21 (36.8%) for both *S. mutans* and *S. sobrinus*, with 12 (21.1%) negative for both *S. mutans* and *S. sobrinus*. 

Table 2 shows the children that were found to have newly acquired mutans streptococci after 1 year and their ages. Four of 22 (18.2%) subjects negative for *S. mutans* at the baseline had acquired *S. mutans* and 15 of 26 (57.7%) *S. sobrinus*. The mean age for the first positive *S. mutans* and *S. sobrinus* result was 60.8 ± 11.4 months and 60.2 ± 8.6 months, respectively, while the age for the first positive *S. mutans* detection ranged from 49 to 71 months old and that for *S. sobrinus* ranged from 49 to 81 months old. 

**Figure 1:**
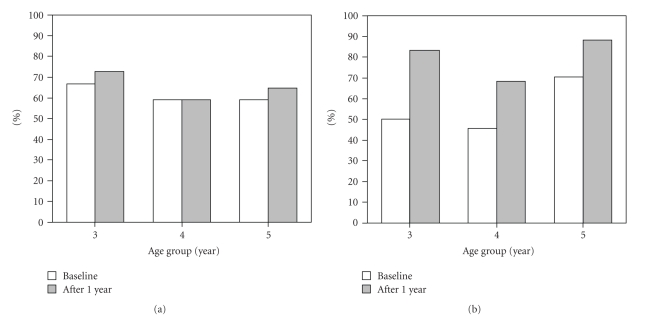
Distribution of *S. mutans* (a) and *S. sobrinus* (b) in different age groups at the baseline and after 1 year.

Figure 1(a) shows the distribution of *S. mutans* in different age groups at the baseline and after 1 year. Overall, the prevalence of *S. mutans* at the baseline and after 1 year was 61.4% and 64.9%, respectively. In the 3-year-old group, 12 (66.7%) of 18 subjects were positive at the baseline and 13 (72.2%) of those were positive for *S. mutans* after 1 year, while the same 13 (59.1%) of 22 subjects in the 4-year old group were found positive at both the baseline and after 1 year. In the 5-year old group, 10 (58.8%) of 17 subjects were found positive for *S. mutans* at the baseline and 11 (64.7%) were positive after 1 year.

Figure 1(b) shows the distribution of *S. sobrinus* in different age groups at the baseline and after 1 year. Overall, the prevalence of *S. sobrinus* at the baseline and after 1 year was 54.4% and 78.9%, respectively. In the 3-year old group, 9 (50.0%) of 18 subjects were found positive at the baseline and 15 (83.3%) were positive for *S. sobrinus* after 1 year. In the 4-year old group, 10 (45.5%) of 22 and 15 (68.2%) of 22 were shown to be positive at the baseline and after 1 year, respectively, while 12 (70.6%) of 17 and 15 (88.2%), respectively, were positive in the 5-year old group. The increased ratios (number of positive subjects after 1 year/number of those at the baseline) for *S. mutans* were 1.1, 1.0, and 1.1, at 3, 4, and 5 years of age, respectively, while the ratios for *S. sobrinus* were 1.7, 1.5, and 1.3, respectively.

## 4. Discussion

We performed a longitudinal study to determine whether mutans streptococci are frequently established in the oral cavities of children during a discrete-time period, known as the “window of infectivity,” that is considered to range from 19 to 31 months of age, with a median age of 26 months [5], and the time of emergence of the primary molars [7, 31]. The majority of studies have suggested that mutans streptococci are not found until teeth erupt and become attachment sites for permanent oral bacterial colonization [5–7], though some authors have mentioned its predentate presence [8–10].

In the present longitudinal study, the PCR method used to detect *S. mutans* and *S. sobrinus* with 16S rRNA primers confirmed the presence of bacteria in all plaque samples (data not shown). This tool provides a more sensitive means of detection of cariogenic bacterial species, as compared with conventional cultural techniques [22, 24, 32]. Further, it has been reported that mitis-salivarius bacitracin inhibits the growth of *S. sobrinus* more than that of *S. mutans* [15, 16], and the recovery of *S. sobrinus* can be significantly underestimated using conventional cultural techniques [33]. We conclude that results of the present study show that this PCR method is suitable for investigation of the intra-oral distribution of *S. sobrinus* as well as *S. mutans*.

The prevalence of *S. mutans* and *S. sobrinus* at the baseline investigation conducted at 3 years old was 66.7% and 50.0%, respectively, and 27.8% for both, which is in agreement with other reports of preschool children [34–36]. The prevalence of children positive for *S. mutans* was constant in each age group at both the baseline and 1 year examinations. It has been suggested that *S. mutans* generally becomes established in the oral cavity of children before the age of 3 years old. However, the prevalence of children positive for *S. sobrinus* after 1 year was higher than that at the baseline, with increase ratios of 1.7, 1.5, and 1.3 in the 3-, 4-, and 5-year-old groups, respectively, indicating that the rate of increase gradually slowed. Further, 57.7% of all the subjects eventually acquired *S. sobrinus* and their mean age for the first positive detection was 60 months, of whom 18.2% had *S. mutans* detected first. It was also noted that *S. sobrinus* was frequently found in the oral cavities of children without *S. mutans* after 3 years of age. Therefore, it is suggested that *S. sobrinus* is established later in the oral cavity of children as compared to *S. mutans* and that acquisition of *S. sobrinus* is still possible after the so-called “window of infectivity.”

In the present study, 7 (50.0%) of the 14 subjects with *S. mutans* became colonized with *S. sobrinus*, while 2 (20.0%) of 10 subjects with *S. sobrinus* were first found positive for *S. mutans*. Considering the means of colonization by mutans streptococci, we speculate that *S. sobrinus* might easily colonize after the colonization of *S. mutans*, allowing it to become established in the oral cavity of *S. mutans* positive children. It is also suggested that the colonization of *S. sanguinis* may have an influence on subsequent colonization by mutans streptococci [37]. Further studies are required to understand the timing of initial infection with *S. mutans* and *S. sobrinus*.

The transmission to and colonization of mutans streptococci in the oral cavity are important factors for the prevention of dental caries. Previous studies have suggested that earlier colonization of *S. mutans* is related to a higher caries risk [34], and that children harboring both *S. mutans* and *S. sobrinus* showed a significantly higher incidence of dental caries than those with *S. mutans* alone in studies that used a conventional cultural [38], indirect immunofluorescence [39], and PCR methods [32]. In addition, the level of mutans streptococci in saliva has been shown to correlate both with past caries experience [40, 41] and future caries activity [42, 43]. We previously reported that incremental caries increases were significantly greater in children with both *S. mutans* and *S. sobrinus*, as compared to those with *S. mutans* alone [4]. Based on the present results, it is predicted that caries risk will increase in 3-year-old children with *S. mutans* already established, since* S. sobrinus* can become established after 3 years of age. We considered that the present results are highly relevant for development of prevention strategies for caries in childhood, as delayed acquisition of mutans streptococci might reduce the number of caries experienced in primary and permanent dentition at later ages [44].

 In conclusion, our results suggest that *S. sobrinus* becomes established later in the oral cavity of children over the age of 3 years old as compared to *S. mutans*.

## Figures and Tables

**Table 1:** Distribution of mutans streptococci at the base line.

*S. mutans*	*S. sobrinus*	Number of subjects (%)
+	−	14 (24.6)
+	+	21 (36.8)
−	+	10 (17.5)
−	−	12 (21.1)

Total		57 (100)

**Table 2:** Children and their ages with mutans streptococci acquired after 1 year.

Organisms present	Age^(a)^	Age range	Number (%)
	(months)	(months)	of subjects
*S. mutans* (− → +)	60.8 ± 11.4	49–71	4/22 (18.2)
*S. sobrinus* ( − → +)	60.2 ± 8.6	49–81	15/26 (57.7)

^(a)^: Mean age ± Standard deviation.
